# Octahedral faceted Si nanoparticles as optical traps with enormous yield amplification

**DOI:** 10.1038/srep08354

**Published:** 2015-02-10

**Authors:** Giovanni Mannino, Alessandra Alberti, Rosa Ruggeri, Sebania Libertino, Agata R. Pennisi, Giuseppe Faraci

**Affiliations:** 1 CNR-IMM, Strada VIII n°5, 95121 Catania, (Italy); 2 Università di Catania, Dipartimento di Fisica e Astronomia, Via Santa Sofia 64, 95123 Catania, (Italy)

## Abstract

We describe a method for the creation of an efficient optical scatter trap by using fully crystalline octahedral Silicon nanoparticles (Si-NPs) of approximately 100 nanometres in size. The light trapping, even when probing an isolated nanoparticle, is revealed by an enormous amplification of the Raman yield of up to 10^8^ times that of a similar Si bulk volume. The mechanism conceived and optimised for obtaining such a result was related to the capability of a Si octahedron to trap the light because of its geometrical parameters. Furthermore, Si-NPs act as very efficient light scatterers not only for the direct light beam but also for the trapped light after it escapes the nanoparticle. These two effects are observed, either superimposed or separated, by means of the Raman yield and by photoluminescence enhancements. The inductively coupled plasma synthesis process performed at a temperature of only 50°C allows for the ubiquitous use of these particles on several substrates for optical and photovoltaic applications.

Radiation amplification in silicon-based optical devices represents an important breakthrough^1^,^2^,^3^,^4^,^5^. So far, most of the attention has been devoted to sub 10 nm sized nanocrystals because, thanks to their quantum confinement capabilities, they are suitable for a wide spectrum of mature applications, such as light emitting diodes, flash memories and flat screens^6^,^7^,^8^,^9^. Nanocrystals also find extremely important applications in biology and medicine^10^,^11^,^12^,^13^ and in most advanced applications^14^,^15^.

The most widely used process for synthesising Si nanocrystals is based on self-assembly from silicon-rich silicon oxide matrices with a subsequent thermal process at temperatures above 1100°C^16^,^17^,^18^. This approach produces spherical nanocrystals and is fully compatible with all established equipment for electronic device fabrication, but it poses severe limitations on the use of compatible substrates, excluding all low cost glass and polymeric substrates.

A completely differently shaped Si-NP formed by SiH_4_ gas thermal dissociation at a high temperature (above 1100°C) has recently been reported^19^. The weakness of this approach is that it only leads to a few irregular crystalline nanoparticles immersed in a mess consisting of a large number of amorphous and polycrystalline particles and again inhibits the use of low cost substrates.

Plasma synthesis is an effective method for producing small nanocrystals. This method is potentially very interesting for its scaling capability and is especially suitable for photovoltaic applications. Indeed, differing from earlier studies^20^,^21^, currently, Si nanocrystals can be produced with accurate control of their size and shape in high-density SiH_4_/Ar plasma mixtures. This approach is very often defined as a non-thermal synthesis of nanocrystals because the substrate is not heated during the plasma treatment or subjected to a subsequent thermal process^22^. Using a plasma it is possible to produce very small nanocrystals with interesting applications and properties^23^,^24^,^25^,^26^,^27^. However, larger Si nanoparticles (Si-NPs) can also be formed in a plasma and do not necessarily have a spherical shape. The most intriguing example of non-spherical Si-NPs is the synthesis of 30-nm-wide cubic Si-NPs^28^,^29^. These large nanoparticles are fully crystalline and are ideal for building innovative Si based single particle transistors.

Of course, the synthesis of NPs with well-defined shapes and sizes is very important. In fact, it is worth noting that in parallel to experimental investigations, many theoretical studies by *ab-initio* or density functional calculations have noted that the Si-NP equilibrium shape is an octahedron^30^,^31^,^32^.

Here, we show a method for the synthesis of octahedral Si-NPs to obtain efficient radiation trapping and scattering with an enormous yield amplification. This effect is realised if the radiation hits a flat planar surface of the nanoparticle and, depending on its shape and refractive properties, enters the nanocrystal and travels back and forth between its walls with an effective path much longer than that of the corresponding bulk crystal. If this geometrical requirement is satisfied, the radiation escaping the nanoparticle adds to the direct light beam, which results in diffuse isotropic Rayleigh scattering, thus producing a very efficient antireflection coating that enhances the surface absorption of the photons on the substrate and on the nanocrystals themselves.

## Results and discussion

**Figure 1:**
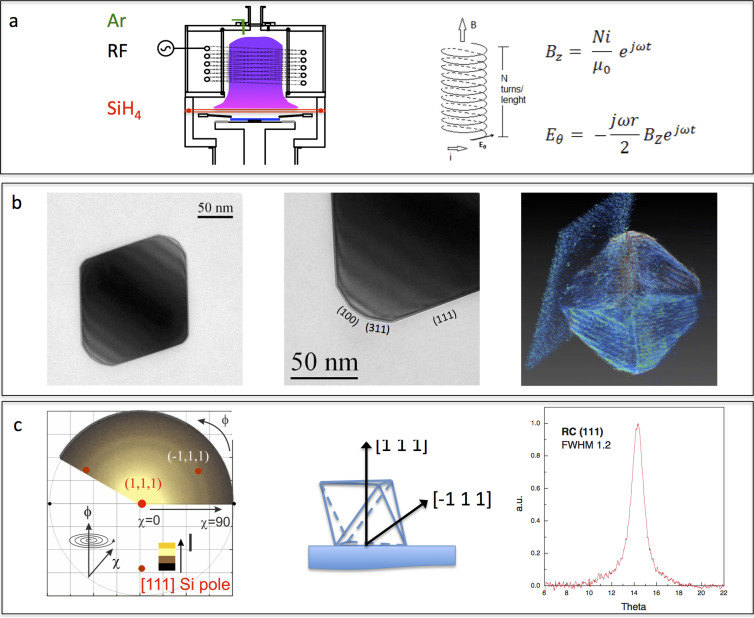
(a) Schematic view of the inductively coupled plasma chemical vapour deposition system with its typical vertical geometry; the RF applied to a solenoid produces a magnetic field perpendicular to the sample and induces a tangential electric field. This scheme, contrary to parallel plate configurations, ensures the plasma is highly ionised (≈5 × 10^11^ ions/cm^3^) and able to form large crystalline Si-NP competitively to Si amorphous deposition, which is very limited; (b) Transmission Electron Microscopy analyses with the indicated planes forming the facets of the Si-NP surfaces. This Si-NP has the (220) zone axis orthogonal to the sample surface to enhance the visibility of the facets. The frame on the right shows a snapshot of a 360° view of a single Si-NP obtained by 3D Transmission Electron Tomography and software reconstruction that shows all the facets of an octahedral Si-NP. (c) X-Ray polar figure, collected at the (111)-Si pole, showing a large signal at χ = 0 and a ring at χ = 70.5°. Those features are related to each other by a crystallographic relationship (i.e., they belong to the same domains) and indicate the presence of a unique zone axis with (111) planes parallel to the substrate. Note, in fact, that the planes at the polar figure centre and those at the ring position form an angle as large as expected in the perfect silicon lattice structure. Those findings attest that most of the particles (86% on the basis of the relative peak intensity, see ref. 33) landed on the substrate with the (111) plane faces exposed upwards, as indicated in the sketch of the octahedral Si-NPs, and touched the substrate with the opposite (111) face. The (111) rocking curve related to those planes is very narrow, which indicates a minimal angular dispersion with respect to the [111] landing axis. The presence of the ring in the polar figure indicates a rotational degree of freedom around the landing axis, as expected, because there is no preferentiality in the x–y directions.

We used an inductively coupled high-density plasma (see Fig. 1a) to obtain monocrystalline, isolated, monodisperse and single shaped Si-NPs. TEM and XRD analyses reveal that the Si-NPs formed during four different short process times (<5 s) are crystalline spheres (see supplemental materials) and that those formed during longer times (>15 s) (Fig. 1b and c) are regular crystalline defect-free octahedrons. The octahedron facets are formed by the (111), (220), (311) and (100) planes as expected from the minimisation of the Woolf energy, although we observed a difference in the relative extension of the planes' surfaces related to the plasma synthesis itself, which is intrinsically a non-equilibrium process, and eventually, the Si-NP size was affected by the same variability^32^,^33^. Almost all Si-NPs (86%) landed on the substrate on their wider (111) facets. An extraordinary clear view of the Si-NPs was obtained by 3D tomography, which unveils unambiguously all the facets of the Si-NPs (Fig. 1b). A complete 3D movie with a 360° rotation is provided in the supplemental material. Last, but not least, X-Ray analyses, reported in Fig. 1c, collected at the (111)-Si pole, show a large signal at χ = 0 and a ring at χ = 70.5° (Fig. 1c). The presence of the ring in the polar figure indicates a rotational degree of freedom around the landing axis, as expected, because there is no preferentiality in the x–y directions. Most particles landed on the substrate with a (111) plane face exposed upwards, as indicated in the sketch (Fig. 1c) of the octahedral Si-NP, and touched the substrate with the opposite (111) face. The total number of particles in this configuration is approximately 86%, calculated on the basis of the relative peak intensity, (see ref. 36).

It is important to note that the synthesis of these octahedral faceted Si-NP was performed at a quasi-ambient temperature (<50°C), making this approach effective, economical, and easily transferable even for deposition on any polymeric substrate. Another advantage of using the plasma synthesis of nanocrystals is that the size distribution is extremely narrow (we estimated less than 3% in size) because the particles are formed in an ambient that is supersaturated with reactive species from the plasma ignition time to the end of the process time, and this ensures that almost all the particles grow at the same rate. This non-conventional deposition produces highly efficient and almost monodisperse “scatter trap” nanoparticles.

**Figure 2:**
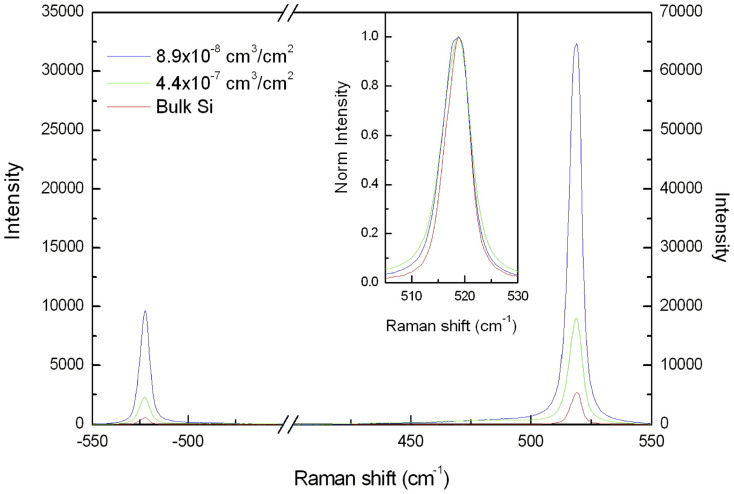
Typical Raman spectra obtained in bulk-Si and for two samples covered with Si-NPs. The cm^3^/cm^2^ unit refers to the volume of Si (cm^3^) contained in the Si-NPs per unit area (cm^2^). The data refer to the total volume of Si-NPs per unit area. A huge signal increase without a shift and/or broadening of the Si peak (see inset) is obtained by the Si-NPs relative to the bulk Si, even though the probed volume is 4 orders of magnitude smaller than that of the bulk Si (≈10^−12^ cm^3^). The ratio between the Stokes and anti-Stokes peaks remains constant from the bulk Si to the Si-NP samples.

The Raman yield as a function of the total deposited Si nanoparticle volume (cm^3^) per unit surface (cm^2^) in isolated Si-NPs has been measured using a He-Ne laser source (632.8 nm) on the TO Raman peak (see Fig. 2). A huge yield—higher than that for bulk Si—was found in Si-NPs for all the depositions and sizes despite their small volumes and scarce substrate coverages. Si-NPs are very wide, with the long axis of the octahedron in the range of 75–160 nm, certainly large enough to be unaffected by the quantum confinement effect, and consequently, no shift and broadening is detected for the Raman peak position (521 cm^−1^) and linewidth (see Fig. 2 inset)^34^. For the same reason, a local increment of temperature due to the laser irradiation is excluded, as attested to by the Stokes-anti Stokes peak intensity ratio^34^.

**Figure 3:**
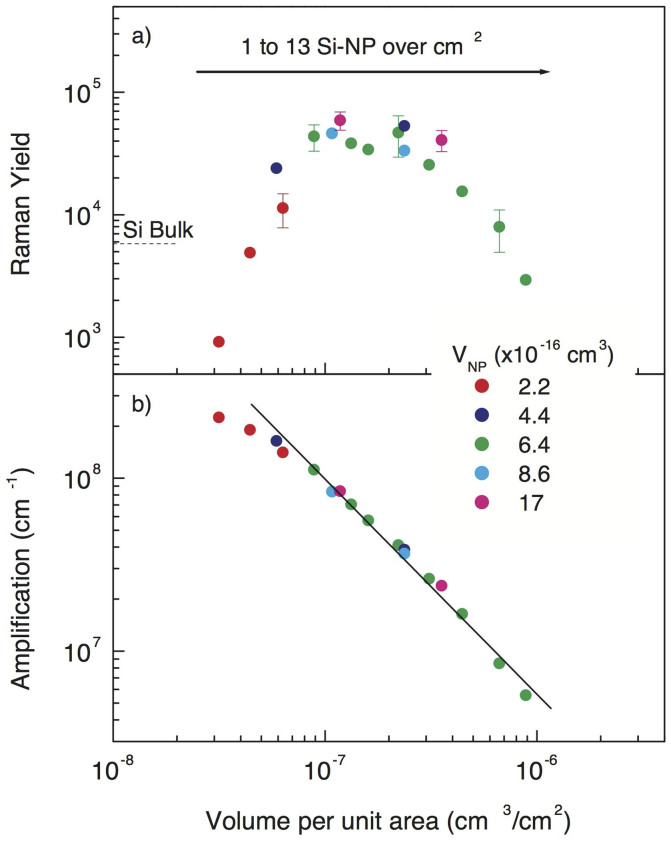
Raman yield (a) and amplification (b) calculated according to eq. (3) in several samples, each covered by Si-NPs with an increasing amount of nanoparticles. The amplification G systematically decreases with increasing Si-NP density for any fixed nanoparticle volume. The solid line in (b) is a linear fit to G as calculated by eq. (3).

The effective Raman amplification factors for isolated Si-NP and for bulk Si are calculated as follows:
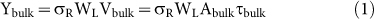

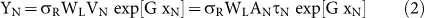
where σ_R_ is the Raman scattering cross section, W_L_ is the laser power density (6 mW/1 μm^2^), V_bulk_ is the probed volume, A_bulk_, τ_bulk_ and A_N_, τ_N_ are the area and path length of the photons in Si bulk or Si-NP, respectively (A_bulk_ = 1 μm^2^, τ_bulk_ = 3.05 μm^35^). From the statistical analysis of thousands of Si-NP of different sizes corresponding to process times of 15, 30, 45, 60 and 120 s, A_N_ is in the range corresponding to diameters of 60 to 120 nm, and τ_N_ is in the range of 45–120 nm. x_N_ = V_N_/(1 μm^2^) is the deposited Si volume per unit surface. From the previous equations, we can obtain the amplification G, defined as:
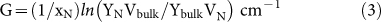


In Fig. 3 we report the Raman yield and the corresponding amplification G as a function of the total deposited Si volume (cm^3^) per unit surface (cm^2^). The figure shows the Raman yield of the deposition process for several sizes of the Si-NP for increasing surface densities in the range of 1–13 nanoparticles/μm^2^, i.e., from very disperse Si-NPs with a density well below complete coverage of the substrate surface up to full coverage. The maximum amplification reaches ≈2 × 10^8^ cm^−1^ and is always greater than 6 × 10^6^ cm^−1^. We note, in Fig. 3b, that the amplification yield is systematically higher for smaller Si-NPs and for more rarefied depositions. The amplification factor G plotted on a log-log scale linearly decreases, confirming the trend expected according to the equation for the description of this trapping phenomenon.

Some saturation at the highest amplification value is observed. Similar results have been obtained using other laser source wavelengths both in the infrared and in the UV spectral range. However, in the latter spectral region, the crystalline Si more easily absorbs the light; therefore, although still present, the amplification has a lower intensity.

**Figure 4:**
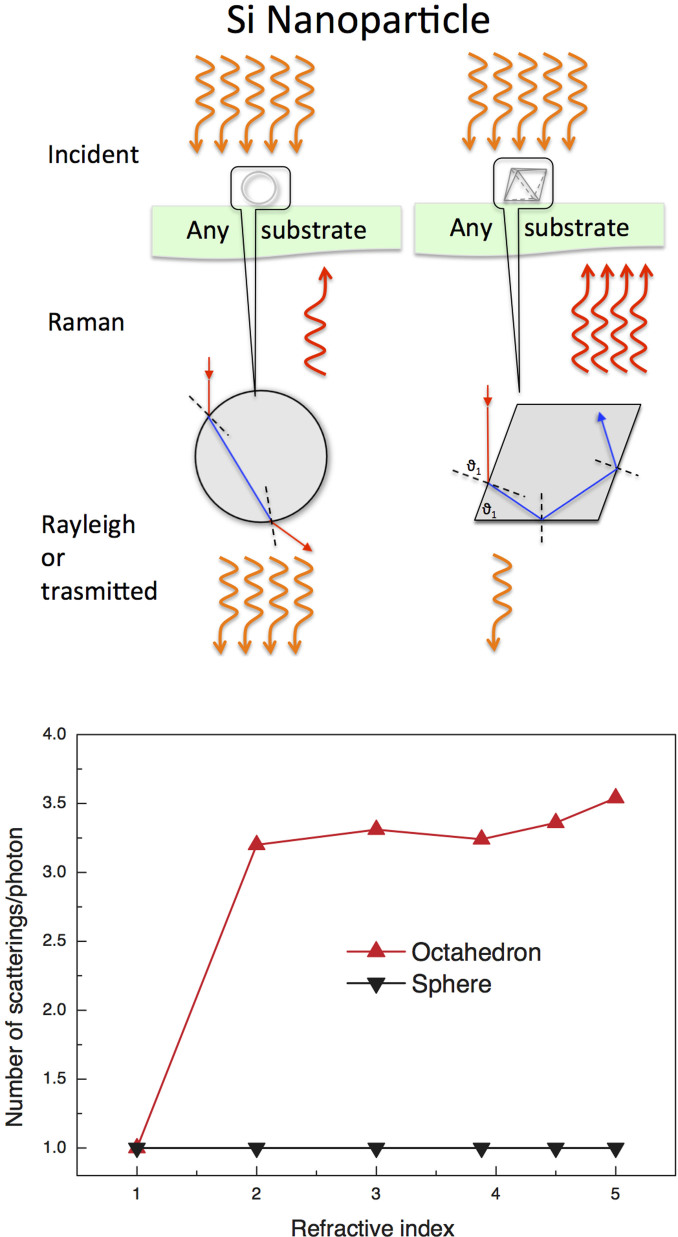
Schematic view of the light trapping mechanism inside the Si-NP at the origin of the Raman signal increase as defined in eq. 3. (top left) In bulk Si, the laser irradiation hits the bulk; Raman photons are emitted according to their cross section, but most of the incident photons are transmitted or undergo Rayleigh scattering; Similarly to a bulk system, in spherical Si-NP, the incident radiation, if not absorbed, exits at the same angle with respect to the radial direction as the incident radiation; therefore, amplification is absent. (top right) In octahedral Si-NPs, the incident radiation hits the Si-NPs with angles of 0° (top facet) or 70.5° (inclined facet), enters the Si-NPs and is efficiently trapped inside. The light travels back and forth inside a very small volume producing, by far, more Raman events, even though the single event cross section remains unaltered. The effective power and volume in the case of Si-NPs are, of course, much smaller than those of bulk Si. (bottom) Simulation of the radiation path inside octahedral and spherical Si-NPs showing a typical enhancement of scattering events in an octahedron as a function of the refractive index.

We argue that the mechanism producing such a high-yield increase is driven mainly by light trapping inside the Si-NPs, as sketched in Fig. 4. A model was developed to explain the huge amplification in terms of a simple coupling of the radiation wavelength with the size of the Si-NPs. We attribute the observed effects to the mechanisms of trapping and reflections between the surface planes of the nanocrystals. In fact, the size of the silicon nanostructures is on the order of one-quarter of the wavelength, allowing a path of the radiation back and forth inside each nanoparticle, with a consequent amplification of the Raman cross section. Actually, the refractive index of crystalline Si for λ = 633 nm radiation is n = 3.88, whereas the extinction coefficient is only k = 0.016.

According to the Mie or Rayleigh cross section as a function of the particle size relative to the wavelength, the photons can be elastically scattered. The scattering cross section is indeed
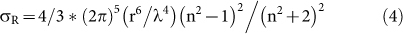
and thus depends on the particle radius r raised to a power for small size particles^36^. Our simulation is performed on a typical and regular Si octahedron with triangular (111) faces at angles of 109.5° or 70.5°. The indices of the eight planes of the octahedron have the equation
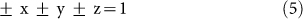


After choosing the incidence angle of the photon relative to the faces of the octahedron, the program uses Snell's Law to calculate the path **p** of the photon inside the crystal before its exit, including the number of total reflections inside of the crystal and accounting for the absorption probability exp(−p/p_d_) along this path, where the penetration depth p_d_ for red radiation is 3.05 μm.

An average should be performed to account for i) the entrance point of the photon; ii) the incidence angle; iii) the refractive index; and iv) the scattering cross section. The critical angle for total reflection according to Snell's Law is 14.9°. Therefore, in a faceted nanoparticle with λ_N_ = λ/n = 163 nm, comparable to the Si-NP size, the red beam entering the nanoparticle cannot easily escape. This happens whenever a photon travels without a loss of energy inside the nanoparticle and hits the surface of the Si-NP in the angular range of 14.9–90°. The ratio between the relative angles is 16.6%, which means that only a small percentage of the photons escape from the crystal without multiple reflections. We performed a simulation of the radiation path inside octahedral Si-NP positioned on the substrate surface, as indicated by XRD analyses. The radiation enters a (111) face of the Si-NP with a specific incidence angle of 0° or 70.5° normal to the (111) facet and is scattered inside according to Snell's Law: n_1_ sinθ_1_ = n_2_ sinθ_2_ (Fig. 4a). Whenever the scattering angle in the NP is lower than the angle of total internal reflection, the photon escapes from the nanoparticle; otherwise it is scattered towards a different face of the crystal, and so on. Our simulation averages over the facets of the NP hit by the photons and demonstrates (see Fig. 4b) a row amplification of the order of that obtained in the experimental data, accounting for the obvious difference between the deep penetration of the photon in the bulk Si and the path of the photons between the surfaces in the Si-NP. As a back test, by reducing the process time we have prepared samples with the same Si volume deposited per unit area but with spherical Si-NP (data not shown). In this case, we did not observe any amplification because a photon entering a sphere with any incidence angle will, if not absorbed, escape the sphere at the same angle, regardless of the incidence point inside the sphere (Fig. 4), and thus this internal reflection phenomenon is not allowed in a sphere.

Other explanations could be proposed but immediately discarded. One could postulate that there is an augmented rate of phonons stimulated by inelastic scattering of photons. We discard this hypothesis because the local temperature does not change significantly. Another possible explanation could be the contribution of electron excitations. In fact, the laser beam in the Si-NP is absorbed either via Raman scattering or via electron excitation. The Raman first-order process produces or absorbs TO phonons (of approximately 63 meV); the electron excitation is possible because the laser photon energy (1.96 eV) is larger than the silicon energy gap for electrons (1.12 eV) and could proceed via indirect phonon-assisted transitions (and/or via impurity levels such as H in the energy gap). Electron excitations produce excitons that decay via non-radiative and/or radiative processes. These two decay paths are, of course, in competition. Whenever the non-radiative process is dominant, phonon production enhances the Raman yield. If the radiative process were to dominate, a photoluminescence production would arise. In our case, we observe enhancement of the Raman yield without a large temperature increase or photoluminescence production in the visible or infrared range. Therefore, this last possibility seems less likely.

Of course, not all the radiation is trapped inside the Si-NP, and the scattered radiation, according to the Rayleigh-Mie theory, can further enhance the Raman yield^37^. A significant portion of the radiation is scattered by the surfaces of the octahedron. This radiation adds to the radiation escaping the nanocrystal and is randomly diffused in the substrate and in the Si-NP, possibly magnifying any physical effects arising in the substrate itself. We argue that we can detect separately the amplifying factors of the different effects produced by the Si-NPs, as a TO Raman peak, or in other specific substrates, e.g., GaAs or a polymeric substrate.

**Figure 5:**
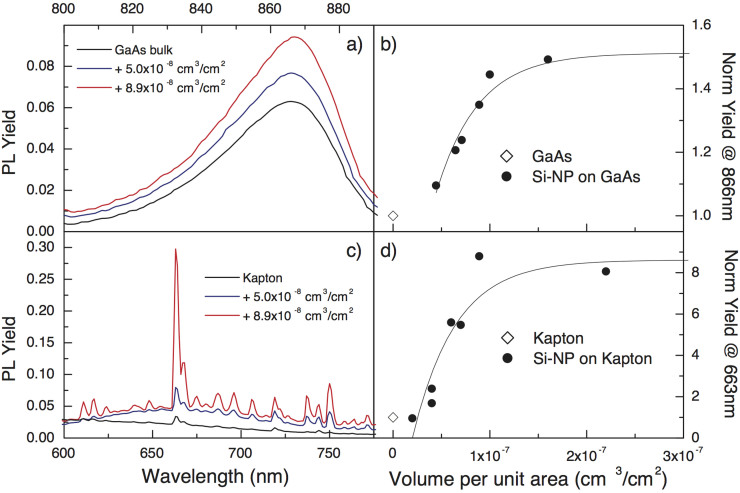
PL spectra (a and c) and PL enhancements (b and d) in GaAs (a and b) and Kapton® (c and d). On the left: PL spectra collected on (a) GaAs and (c) Kapton® layers covered by Si-NPs compared to the reference PL spectrum in a reference bulk sample of each material. On the right: the normalised yield as a function of the increasing volume of Si constituting the nanoparticles per sample unit area. The measurements on Kapton® layers were performed in transmittance to avoid any contribution from the holder to this semi-transparent substrate. The increased yield in the samples covered by Si-NPs is evident in both materials, but the extent of the amplification is much higher in a Kapton® layer—up to a factor of 10—than in the GaAs layer. The solid line is an exponential fit to the data. The fitting curves have the same decay constant and differ only in amplitude, as discussed in the text.

To test the Si-NP activity on a “traditional” substrate, the same depositions used to test the Raman amplification were performed on a GaAs substrate. Let us first use a GaAs substrate for the Si-NP deposition, comparing the PL due to laser irradiation (1.96 eV) with or without the Si-NP layer. The direct interband electron transitions in GaAs with a gap 1.4 eV are followed by PL decay. Of course, no PL contribution is attributed to the Si-NP, but as visible in Figure 5a, the GaAs PL is amplified relative to that of the uncoated GaAs wafer. The PL yield enhancements are summarised in fig. 5b as a function of the Si-NP deposited volume per unit surface.

With the intention to generalise the previous PL amplification effect to any luminescent substrate, a corroborating experiment was performed using a Kapton® thin film. This material emits a multi-peak PL spectrum according to the measurements shown in Fig. 5c, possibly due to structural defects or, more likely, to an optical fingerprint of the polymeric chains. In fact, the same optical markers, with the same relative intensities, were detected in all locations of the probed sample region. When a deposition of Si-NP is performed on such a polymeric layer, we observe an enhancement of PL yield for all the peaks relative to that of the uncoated substrate, as shown in Fig. 5c. Again, no PL contribution can be attributed directly to large Si-NPs, which could eventually absorb the radiation. In this case, an enhancement due to the presence of the Si-NPs is observed, similar to the enhancement observed for the GaAs samples, with a total enhancement factor as high as 10. The amplification mechanism can be easily delineated. In the uncoated substrate, the radiation is either scattered or absorbed for excitation and consequent PL decay with a known cross section. In the NP-deposited substrate, the particles act as strong capture and scatter centres. Impinging photons might be trapped by the NPs before hitting the surface, and, because of the internal reflection, be reemitted with a strong change in the angle of incidence, or they can be scattered at high angles; in either case, the radiation path length will be greatly increased, with a consequent observable amplification. Of course, when the Si-NP coverage increases beyond a certain level, the PL yield is strongly reduced because of the absorption.

Looking more carefully at the functional dependence of the enhancement factors with the Si volume deposited per unit area, we can identify a common curve describing the range of amplification as:
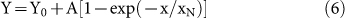
where Y_0_ is the uncovered substrate reference yield, A is the specific enhancement factor, and x_N_ is the saturation nanoparticle volume per unit area.

From the fits of fig. 5b and d, we obtain the following: for both curves, an identical exponential trend which saturates at x_N_ ≈ 2 × 10^−7^ cm^3^/cm^2^, as occurred for the Raman yield (see Fig. 2a); the A values change considerably from 0.075 for GaAs up to 0.46 for Kapton. Therefore, the A value depends on the emission rate of the particular substrate material. Thus, regardless of the specific extent, the PL enhancement is a unique phenomenon related to the underlying material driven by the presence of the Si-NP, wherein the trapping and scattering effects cooperate and are the highest when the particles are relatively distant from each other.

In summary, a viable simple route for obtaining yield amplification by appropriate coupling of the device shape with the radiation wavelength of (but not limited to) 632.8 nm is reported here. We demonstrated the existence of an amplification mechanism that involves trapping the radiation in such a way that the photon travels back and forth between the walls of the nanostructure like the light in a sparkling gemstone. In other words, assuming a defined cross section for a physical effect, such as the Raman scattering or any other similar interaction, it is possible to tremendously increase the final rate, thus obtaining a high amplification simply by making the path of the radiation longer and longer. At the same time, this nanoparticle shape can be profitably used as an antireflective coating, for example, in photovoltaic processes in which a low temperature fabrication process is mandatory, such as for polymeric substrates. The use of nanoparticles could replace complex and expensive processes such as mechanical or chemical patterning. The fabrication of such scatter traps that are sufficiently large to be manipulated and positioned is accomplished by realising a design and shape of the nanostructure that has surface walls along planes acting as mirrors for the trapped radiation. We demonstrated that this novel nano-device is a real milestone towards the fabrication of simple and cost effective devices and that it opens the route to unexplored possibilities not only for other Si-NP shapes but also for other group IV nanoparticles^38^,^39^, with several applications in the optics and photovoltaic fields.

## Methods

We used an inductively coupled high-density plasma (see Fig. 1a) to properly obtain monocrystalline, isolated, monodisperse and single shaped Si-NP. Samples are prepared in vacuum (5 × 10^−7^ Torr) in an inductively coupled plasma chemical vapour deposition reactor in a way that exploits the capability of this “non-thermal” plasma to enhance crystalline Si-NP formation rather than SiH_4_ decomposition into amorphous Si deposition. This reactor has a vertical geometry, the plasma is highly ionised (2 ÷ 6 × 10^11^/cm^3^), and the substrate is maintained at a low temperature (50°C). The process pressure is in the 20–23 mTorr range. The deposition is performed on a circular area with a diameter of 6 inches. The density of the particles has been maintained as low as possible (0.1 × 10^7^/cm^2^), and it is increased by performing the process for a specific time and repeating the process as many times as are needed to achieve the desired density. The process times used are 15, 30, 45, 60 and 120 s. Silicon nanoparticles were deposited on different substrates, namely quartz, glass, GaAs wafers and Kapton® foils.

The samples were analysed by transmission electron microscopy (TEM) using a JEOL JEM 2010 microscope with a LaB6 thermionic source, operating at an acceleration voltage of 200 kV and equipped with a Gatan multi-scan digital camera. All the analyses were carried out in the bright field condition and some in the high-resolution mode. The TEM tomography was acquired using a JEOL JEM-ARM200F microscope operating at an accelerating voltage of 200 keV and equipped with a cold FEG emitter and a single tilt sample holder with a tilt range up to +/−50° for acquisition. Full 3D reconstruction and visualisation are obtained using dedicated software.

X-ray diffraction analyses (XRD) are performed by using a high angular resolution D8Discover Bruker AXS diffractometer equipped with a Cu K_α_ source, a Goebel mirror and long Soller slits. Polar figures were collected by selecting the (111) pole of the cubic silicon structure at 2θ = 28.44° with the intent of investigating the eventual texturing in the statistical distribution of the most intense silicon planes. For further support, rocking curves were also collected to measure the spread of the landing axis.

The samples are investigated by micro Raman spectroscopy for detecting the transverse optical (TO) vibrational peak situated at 521 cm^−1^ for bulk Si at room temperature (300 K). Raman spectra are collected in the backscattering geometry with a HORIBA Jobin Yvon system equipped with an Olympus BX41 microscope. He-Ne laser radiation at a wavelength of 632.8 nm is focused to a spot size of the order of 1 μm by a 100× objective. The laser power on the sample is 6 mW, and a 550 mm focal length spectrometer with 1800 lines/mm grating is used for collecting the Raman spectra.

PL measurements were performed by illuminating the sample with two different laser sources: at 405 nm (using a Coherent Cube laser pulsed by an internal trigger) and at 633 nm (using a Thorlabs HeNe laser pulsed by an optical chopper). The sample was placed on a special holder with a hole where the laser impinged to avoid the introduction of artefacts due to the collection of the light reflected by the holder surface. The light reflected by the sample was focused, using two optical lenses, on the input slit of a monochromator (Digikrom DK480) and detected by a Photomultiplier Tube (PMT, Hamamatsu R943-02) placed on the monochromator's output slit. The signal was amplified by a lock-in amplifier (Stanford Research) and recorded by a PC.

## Supplementary Material

Supplementary Information TEM Tomography

Supplementary Information Supplementary material caption
