# Supplementary material for: Octahedral faceted Si nanoparticles as optical traps with enormous yield amplification
Source: Sci Rep. 2015 Feb 10;5:8354. doi: 10.1038/srep08354 (PMC5389026; doi:10.1038/srep08354)
Supplement: Supplementary Information — Supplementary material caption [file srep08354-s2.doc]

**Supplementary Material**

**Octahedral faceted Si nanoparticles as optical trap with enormous yield amplification**

Giovanni Mannino^1,*^, Alessandra Alberti^1^, Rosa Ruggeri^1^, Sebania Libertino^1^, Agata R. Pennisi^2^ and Giuseppe Faraci^2^

1 CNR-IMM, Strada VIII n°5, 95121 Catania (Italy)

2 Università di Catania, Dipartimento di Fisica, Via Santa Sofia 64, 95123 Catania (Italy)

*Legend*

*3D Transmission Electron Tomography and software reconstruction which shows a 360° view of all the facets of an octahedral Si-NP.*
